# The Effect of Self-Reported Lactose Intolerance and Dairy Consumption on Bone Mineral Density among American Hip Arthroplasty Patients: A Cross-Sectional Study

**DOI:** 10.3390/ijerph17197182

**Published:** 2020-09-30

**Authors:** Nikola K. Hamilton, Omorogieva Ojo, Amanda Rodrigues Amorim Adegboye

**Affiliations:** 1 School of Life Sciences, University of Westminster, London W1W 6UW, UK; Hamilton.nikola@gmail.com; 2 School of Health Sciences, Faculty of Education, Health and Human Sciences, University of Greenwich, London SE9 2UG, UK; 3 School of Human Sciences, Faculty of Education, Health and Human Sciences, University of Greenwich, London SE10 9LS, UK; A.Adegboye@greenwich.ac.uk

**Keywords:** lactose intolerance, osteoporosis, bone mineral density, dairy intake

## Abstract

The relationship between osteoporosis and lactose intolerance is unclear. This study aims to evaluate the association between self-reported lactose intolerance and symptom severity caused by lactose malabsorption and bone mineral density (BMD). A total of 496 American hip arthroplasty patients took part in this study. Information on BMD and socio-demographic factors were retrospectively extracted from medical records. BMD of the lumbar spine (LS), femoral neck of the operative hip (FNOH), and femoral neck of the non-operative hip (FNH) were measured via dual-energy x-ray absorptiometry scans (DXA). Patients also completed a survey regarding dietary and lifestyle habits from the time of surgery. We found that 9.3% of participants reported lactose intolerance and 33.3%% suffered from either osteopenia or osteoporosis in at least one (location). The population that did not self-identify as lactose intolerant consumed significantly more dairy (*p* < 0.0001) and animal protein (*p* = 0.004) than those with intolerance. There was no significant difference in BMD between self-identified lactose intolerant individuals and non-lactose intolerant individuals. In a stepwise multiple regression analysis, body mass index (BMI) and age were the only common predictors of BMD for all locations (*p* < 0.05). However, yogurt intake was a significant predictor of BMD of FNOH in the multivariate analysis. This study suggests that lactose intolerance is not associated with bone mineral density. We also found that being vegan or vegetarian may increase the risk of low BMD.

## 1. Introduction

Osteoporosis is a skeletal disorder defined by bone mass degradation [1], creating irreversible changes in bone structure and increasing fracture vulnerability [2]. Osteoporosis is a silent disease [3] that does not elicit symptoms before a fracture occurs [1]. Worldwide, nearly 200 million people are affected by low bone mass, attributing to 8.9 million fractures each year [3]. By the year 2050, fracture rates are expected to increase by 240% in women and 310% in men [4]. Consequently, 5964 disability-adjusted life years (DALYs) were lost in North America and Europe due to hip fractures [5], attributable to 0.21% of the total global burden of disease [6]. The financial and social burden posed by this condition impels a clear understanding of the risk factors involved in its development so that preventative measures may be implemented.

Dual-energy X-ray absorptiometry (DXA) scans are the most widely validated means for measuring bone mineral density (BMD) [7]. As BMD declines, the risk of fragility fractures increases incrementally. Thus, parameters for osteoporosis and osteopenia (low bone mass, but to a lesser extent than osteoporosis) are typically based on BMD [8].

Calcium consumption has been promoted to prevent and treat osteoporosis [9]. In the USA, nearly three-quarters of dietary calcium is derived from dairy [10]. However, on a global scale, calcium consumption and hip fracture rates appear to be inversely related [11,12]. While developing nations consume less dairy [13], they also experience fewer hip fractures [12,14]. Communities consuming more calcium suffer from more osteoporosis [15]. African Bantu women, for instance, consume on average one-third of the USA recommended dietary allowance of calcium for adults (1000 mg/day) [16], without dairy or calcium supplementation, and exhibit the lowest incidence of osteoporosis in the world. However, differences in sunshine exposure, physical activity, and other aspects of lifestyle exist between Western and African populations, particularly in rural areas.

While genetics may account for over 50% of bone density [17], the remainder is dependent on environmental factors such as exercise, hormonal status, and/or dietary factors [18] including protein, fibre, iron, alcohol, sodium, potassium, phosphorous, magnesium, fluoride, and vitamins A, B, C, and K [18]. Thus, the paradoxical relationship between high calcium intake and heightened osteoporosis prevalence is likely the result of many intertwined factors.

Vitamin D, produced via skin exposure to sunlight, is essential for calcium absorption. Variations in vitamin D availability could potentially confound migration studies and/or other analyses of diet and fracture rate. Long-term, excessive calcium consumption may also impair calcium and vitamin D regulatory systems [12]. This risk might be especially dangerous for populations consuming high levels of animal protein [11,12,19,20]. As animal protein is digested, it stimulates acid release from the stomach. To buffer the corresponding metabolic acid load, this, in turn, enhances calcium excretion. Whether the amount of calcium retained in the body overrides the amount excreted is controversial. Some research has documented a net calcium loss alongside animal protein consumption [21], while other studies have indicated that, regardless of urinary excretion, bodily stores of calcium may not be affected [22] or may even improve [23].

Intake of plant protein does not seem to increase urinary calcium excretion [20]. Globally, the greater the ratio of animal protein to plant protein consumption, the greater the incidence of fractures [19]. Furthermore, calcium absorption rates are affected by the combination of all available nutrients (e.g., animal protein, sodium, potassium levels, etc.). Therefore, analyzing calcium content alone (of specific food or a diet) is insufficient to determine bioavailability. Recommended daily allowance of calcium may vary greatly depending on the specific calcium source and overall diet composition. For instance, while sodium consumption enhances urinary calcium excretion, increasing dietary potassium may counteract this, particularly in postmenopausal women [24].

As dairy is a rich source of calcium, animal protein, and often sodium, its effects on osteoporosis are unclear. Lactose intolerant individuals (LIIs) in the USA, who typically avoid dairy, but otherwise follow a western diet [25], may help clarify this trend. LIIs lack the enzyme lactase, responsible for break lactose into two monosaccharides, glucose and galactose, before being absorbed across the intestinal mucosa [26]. Without lactase, lactose is passed into the large intestine, where it is digested via bacterial fermentation causing abdominal cramps, flatulence, vomiting, diarrhoea, and/or audible bowel sounds [27].

Most people are lactase secretors at birth, but lose this ability at some point during maturation. Persistence of lactase secretion throughout adulthood varies greatly [11], creating a variable degree of lactose intolerance throughout the world [28]. The prevalence of LIIs in the USA varies greatly according to ethnic groups: 10–15% of Caucasians, 65–75% of African Americans [29], 60% of Hispanics, and 100% of Asian-Americans and Native Americans [10]. However, the available literature is unclear regarding the effects of lactose intolerance on bone health [25]. While some studies have demonstrated positive relationships between lactose intolerance, osteoporosis [10], and low BMD [30,31], other studies have found no significant associations between lactose intolerance and BMD [10,25,32].

This study aimed to add to the existing body of literature regarding bone health and lactose intolerance by assessing whether lactose intolerance is associated with low BMD due to lower dairy consumption, and whether the severity of symptoms, caused by lactose maldigestion, affects BMD among LIIs.

## 2. Materials and Methods

### 2.1. Study Design and Population

This was a cross-sectional study of adult patients who underwent hip arthroplasty at a private surgery centre in the United States (Columbia, South Carolina) between 2003 and 2017, with available demographic data and DXA scans in a computerised medical records database and retrospectively completed a questionnaire on symptoms of lactose intolerance, dietary intake, and lifestyle factors at the time of the surgery.

Participants were selected via convenience sampling, based on the availability of BMD scores at a surgery center in the United States. All patients received hip arthroplasty surgery (either total hip replacement or hip resurfacing). Hip resurfacing is typically performed on younger patients with severe degenerative hip arthritis/osteoarthritis (approximately 70%), but also hip dysplasia, osteonecrosis, and post traumatic arthritis [33]. The BMD of osteoarthritic patients may be greater than the general population [34]. Patients aged <18 years or with missing or inaccurate contact information, missing BMD t-scores, and/or death were excluded. Patients who received bilateral hip arthroplasty were only analyzed in respect to their first operation.

**Figure 1:**
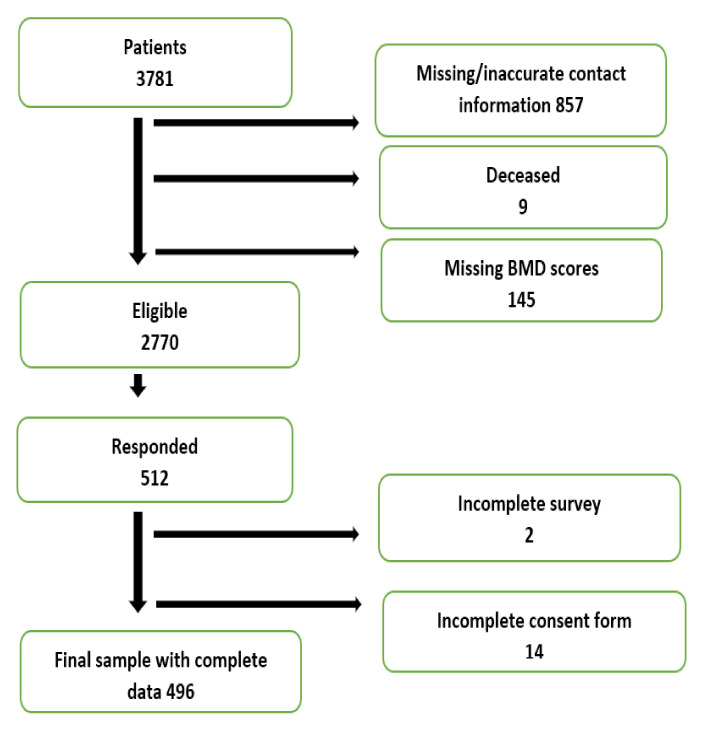
Flow chart of participants’ recruitment.

In total, 3781 adult patients (71% male, 29% female) were identified and 2770 were deemed eligible to participate in the study (Figure 1). A survey, a study information sheet, and informed consent were sent to all eligible patients. When email addresses were available, participants received electronic questionnaires (*n* = 1370, 49.46%). All other participants were contacted by mail (*n* = 1400, 50.54%). Out of 2770 invited to participate in the study, 496 completed the survey and informed consent.

A sample size of 496 participants yielded a power of 0.978, deducing that sample size was adequate and the likelihood of a type two error was small. The power analysis was performed in G*Power software for Macintosh OS/OS X (version 3.1.9.2; Heinrich-Heine-University, Düsseldorf, Germany). 

### 2.2. Data Collection

The survey inquired about dietary habits, exercise, menopausal status, medications, vitamin supplementation, and lactose intolerance at the time of hip arthroplasty surgery. Calcium supplementation, use of digestive aids, cardiovascular exercise, and strength-building exercise were recorded in intervals (<1 time weekly, 1–3 times weekly, 4–7 times weekly, and >7 times weekly). The survey included broad questions on intake of milk, cheese, yogurt, egg, meat/poultry/fish, calcium-fortified products, and lactose-free milk consumption. The frequency was recorded at similar intervals (<1 time weekly, 1–3 times weekly, 4–7 times weekly, and >7 times weekly). Dairy consumption was estimated as a sum of all dairy (milk/butter, cheese, and yogurt) intake, ranging from 3 to 12. For example, a score of 3 represented <1 weekly serving of each dairy product, and a score of 12 represented >7 weekly servings of each dairy product. Animal protein consumption was estimated as a sum of meat/poultry/fish, egg, and dairy intake, ranging from 5 to 20. For example, a score of 5 represented <1 weekly serving of each animal product, and a score of 20 represented >7 weekly servings of each animal product.

Participants were asked whether they had been diagnosed with lactose intolerance by a physician. Lactose intolerance was self-reported and not confirmed by hydrogen breath tests or prove of physician diagnosis. The severity of lactose intolerance was measured on a visual analogue scale, measuring 5 items: flatulence, diarrhoea, vomiting, abdominal cramping, and audible bowel sounds. The scale ranged from no symptoms (0) to maximum symptoms (10), based on a validated lactose intolerance questionnaire [27]. The length of lactose intolerance and calcium supplementation were recorded in intervals (<1 year, ≥1 but <5 years, and ≥5 years). Multiple choice and open-ended questions inquired about the active pursuit of dietary calcium.

The medical records database was established preoperatively. BMD of the femoral neck of the operative hip (FNOH), the femoral neck of the non-operative hip (FNH), and/or lumbar spine (LS) were measured by DXA scans. T-scores were used as a point of comparison amongst participants [35], following WHO guidelines for osteoporosis (t-scores ≤−2.5) and osteopenia (t-scores between −1 and −2.5) [36].

Preoperative weight and height were extracted from the medical database and used to calculate body mass index (BMI, kg/m^2^). Patients were classified as underweight (<18.5), normal-weight (≥18.5 and <25), and overweight (≥25 and <30) and obesity (≥30) according to the WHO (2017) BMI cutoff points. Residential latitude, based on patients’ zipcode, was determined by the [37]. Participants who live at/above 37°C or at/below −37°C latitude were referred to as at-risk latitude (ARL), denoting the decreased availability of vitamin D at such localities [38]. All patients who lived within −37 and 37 degrees latitude were referred to as no risk latitude. Latitude was used in this study as a proxy of sun exposure at the time of the surgery.

### 2.3. Ethics

The study followed ethical guidelines established by the Health Insurance Portability and Accountability Act of 1996 and The Data Protection Act of 1998. The project was approved by ethics committees at both Providence Hospital (United States) and the University of Westminster (UK reference code ETH1617-0769). All patients were provided with a patient information sheet and signed a letter of consent. Patient information sheets included a phone number and email address for questions and/or clarification, as needed.

### 2.4. Statistical Analysis

The general characteristics of the study population were reported as mean ± SE for continuous variables and absolute (*n*) and relative frequency (%) for categorical variables. Lactose intolerance was analyzed as a binary variable (yes/no). The severity of lactose intolerance was measured as a continuous variable, with the sum of all symptoms ranging from 0 to 50 [27]. Poor bone health (PBH) was defined as T-scores < −1.0 (inclusive of osteoporosis and osteopenia). The Shapiro–Wilk test was used to test for normality.

Pearson’s chi-square test and unpaired t-test were employed to detect differences in socio-demographic factors, BMI, BMD, dietary intake, use of dietary supplements, and menopausal status (only for women) between lactose intolerant and non-lactose intolerant participants. One way ANOVA was performed to test differences in BMD of the FNOH, FNH, and LS according to the general characteristics of the study population. A significance level of alpha <0.05 was used for comparison between groups. Turkey’s multi-comparison test was employed as a post-hoc analysis.

To avoid loss of information associated with categorization, dependent variables (BMD of the FNOH, FNH, and LS) were analyzed as continuous. Multiple linear regression was performed to identify predictors of BMD. All available covariates that could influence BMD, according to current literature, were explored, including calcium supplementation, lactose intolerance, gender, BMI, the sum of all dairy and protein consumption, and latitude. Variables were included in the model when they were correlated with the dependent variables (BMD of the FNOH, FNH, and LS) in the bivariate analysis (*p* < 0.20). With these variables, a stepwise multiple linear regression analysis was performed. Associations were considered significant in the final model when the *p*-value was <0.05. Within the lactose intolerant population, the severity of symptoms was analysed with a linear regression model. A significance level of alpha <0.05 was used to measure the strength of this relationship. SPSS^®^ (version 24; IMB corp., Chicago, IL, USA) was used for all statistical analyses.

## 3. Results

Table 1 shows the general characteristics of the study population of 496 patients who had hip arthroplasty according to lactose intolerance status. In total, 68.9% (342) of the study population were male, 9.3% (46) reported lactose intolerance and 33.3% (165) had either osteopenia or osteoporosis in at least one location (Table 1).

Of the lactose intolerant population, only 6.5% (3) reported a physician diagnosis of lactose intolerance; 65.2% (30) were males, 13% (6) reported taking vitamin D supplements, and 24.3% (9) reported taking calcium supplements (Table 1). Individuals with lactose intolerance did not significantly differ from those without lactose intolerance regarding most of the variables assessed with the exception of vitamin D supplementation, consumption of food fortified with calcium, dairy, and protein. Individuals without lactose intolerance were more likely to take vitamin D supplements and consume food fortified with calcium at least once a week and report a higher intake (sum of frequency score) of dairy consumption and animal protein.

Table 2 shows the mean bone mineral density (BMD) (t-score) for femoral neck of the operative hip (FNOH), femoral neck of the non-operative hip (FNH), and lumbar spine (LS) according to population characteristics. There was a trend toward lower BMD among women compared to men. However, the results were only significant for the lumbar spine. Postmenopausal women have significantly lower BMD than pre and perimenopausal women in all three BMD indicators (FNOH, FNH, and LS). Mean BMD did not differ according to the consumption of calcium supplement, foods fortified with calcium, milk, cheese, reporting cardiovascular exercise, and living in at-risk latitude.

Individuals who reported consumption of vitamin D supplements tended to have higher BMD means. However, the results were significant (*p* < 0.05) for FNOH only. Individuals who reported consumption of yogurt more than once a week presented significantly higher BMD FNOH compared to those who did not consume yogurt or consume less than once a week (*p* = 0.015). Results for FNH and LS were not statistically significant.

Lower BMD for femoral neck of the operative hip, femoral neck of the non-operative hip, and lumbar spine among vegetarian and vegan individuals was observed compared to those who reported consumption of animal foods. The results reached statistical significance for LS (*p* <0.001) and FNOH (0.049) and presented borderline *p*-values (*p* = 0.068) for FNH. Mean BMD significantly varied across BMI groups in all three indicators (FNOH, FNH, LS). The higher the BMI category the higher the mean BMD (Table 2).

### 3.1. Analysis of the Lactose Intolerant Population

The average length of lactose intolerance was 2.43 ± 0.82 years. The average severity of symptoms, when consuming lactose, was 18.31 ± 10.89. As presented in Table 3, the average BMD did not significantly differ between those with or without lactose intolerance. In the bivariate analysis among those who have lactose intolerance (*n* = 461), there was a trend towards a positive relationship between the severity of lactose intolerance symptoms and BMD, but the results were not statistically significant for FNOH and FNH. BMD of LS was the only indicator that reached statistical significance in the linear bivariate analysis between the score of severity of symptoms and BM (Coefficient = 0.06, SE 0.02, *p* = 0.047). When this model was adjusted for intake calcium supplement, the association was no longer significant (results not shown).

### 3.2. Predictors of Bone Mineral Density

In a stepwise multiple linear regression (Table 4)*,* BMI and age were the common predictors of BMD for all three indicators. BMI was positively associated with BMD while age was negatively associated with BMD. Consumption of yogurt was positively associate with BMD FNOH, while cardiovascular exercise was positively associated with BMD FNH. For LS, gender and being vegan or vegetarian were also significant predictors.

## 4. Discussion

This study aimed to analyse the difference in BMD according to lactose intolerance status while accounting for various dietary and lifestyle factors and to determine whether the severity of lactose intolerance symptoms affects BMD. The results demonstrated that BMD did not differ significantly between lactose intolerant and lactose tolerant individuals, albeit greater consumption of animal protein and dairy by lactose tolerant individuals. The results also showed that being vegan or vegetarian is associated with lower BMD of the lumbar spine. These results concur with several previous studies. In a study of 34 lactose intolerant individuals (LIIs), Alhava et al. [39] found that participants consumed significantly less milk and dietary calcium than the normal population, however, the osteoporotic risk did not differ from previously established normal values. In another study of 218 individuals, Kudlacek et al. [25] found no significant difference in BMD between LIIs (*n* = 115) and non-LIIs (*n* = 103). However, this study established lactose intolerance based on physician diagnosis alone (via a hydrogen breath test) and not all LIIs demonstrated clinical symptoms of intolerance. It is, therefore, possible that calcium consumption did not differ between groups.

In a study of 58 post-menopausal Italian women, Corazza et al. [30] also found that lactose intolerance did not directly influence BMD and that calcium intake and symptom severity were inversely related. This would suggest that the more severely individuals experienced symptoms of intolerance, the less dairy he/she consumed. Similarly, in the Kudlacek et al. [25] study on BMD and self-reported symptoms of lactose intolerance, no significant differences were found between BMD of the lumbar vertebrae and nondominant distal radius. However, of the 115 lactose intolerant individuals, 17% reported no symptoms of intolerance. Kudlacek et al. [25] deduced that individual perception of symptomology may be a better predictor of BMD than clinical diagnosis, as patients with severe intolerance are expected to avoid dairy. In the current investigation, we found that within the lactose intolerant population, those who experienced the most severe symptoms of lactose intolerance demonstrated stronger BMD of the lumbar spine. No relationship was apparent in the FNH or FNOH.

The Austrian study [25] previously reported no significant difference in BMD between the lactose intolerant individuals and non-LIIs. Furthermore, Slemenda et al. [40] also employed hydrogen breath tests to analyse lactose intolerance. In a sample of 342 adult, female twins, data showed no significant correlation between lactose intolerance and BMD of the femoral neck or midshaft radius. However, physician-diagnosed lactose intolerance does not always manifest with symptoms [25,41]. In both studies, hydrogen breath test diagnoses were used for some individuals who were unaware of their status. Therefore, the participants may have consumed dairy without any symptoms of intolerance and could have consumed unlimited dairy products. By analysing self-reported lactose intolerance, versus hydrogen breath test diagnoses, the current investigation may have a heightened sensitivity to self-imposed dairy restrictions.

In the stepwise multiple regression analysis, the amount of dairy consumption was not a significant predictor of BMD. We only found a significant association between yogurt intake and BMD of FNOH. The inaccuracy of retrospective food frequency questionnaires may have underestimated this association. Lactose intolerance symptoms may, therefore, be a more accurate depiction of dairy avoidance.

As the current investigation was based on self-reported lactose intolerance, all lactose intolerant individuals experienced symptoms of maldigestion. Thus, findings that the non-lactose intolerant population consumed significantly more dairy than the lactose intolerant population are logical. Many studies conclude that by reducing dairy intake, calcium intake simultaneously decreases [9], placing lactose intolerant individuals at heightened risk of osteoporosis [42]. Although there was significantly less dairy consumption by lactose-intolerant individuals, no significant difference in BMD was observed in this study between lactose intolerant individuals and non-LIIs.

Additionally, the majority of research that correlates lactose intolerance to low BMD attributes this relationship to a reduction of dietary calcium intake [31,32]. Other studies also describe this relationship to the absence of lactose [43], since lactose increases calcium diffusion and may osmotically alter junctions between epithelial cells [44]. However, these reports require further investigation [31,45].

According to Weaver et al. [9], adequate levels of calcium may be attained from plant-based sources, though it is less concentrated and therefore more practical to consume dairy. However, it is well documented that food is more than the sum of its parts [46]. Nutrients, such as calcium, cannot be analysed in isolation, as compounds often behave differently depending on the synergistic or pernicious effects of the composite [46].

In addition to high levels of dietary calcium, dairy products are often high in animal protein and sodium, both of which enhance urinary calcium excretion. This may explain why calcium is more readily absorbed from green vegetables and legumes (40–64%) than from dairy (32%) [47]. Therefore, rather than recommending dairy to support bone health, plant-sourced calcium may be superior [48,49,50,51,52]. Different dietary patterns (e.g., plant-based vs. meat-rich diets) may also necessitate different thresholds of calcium, potentially explaining why nations consuming the most animal protein suffer from the greatest incidence of fractures [19,20]. However, in the present study, we found that being vegan or vegetarian was negatively associate with BMS of the lumbar spine. However, this study lacks comprehensive habitual dietary data, detailed information of dietary supplementation, and blood levels of calcium and vitamin D to clarify if this association was mediated by factors related to the quality of the diet and specific macro and micronutrients.

The findings from this study demonstrated that non-LIIs consumed significantly more animal protein than lactose intolerant individuals. However, neither group differed in egg, fish, poultry, or meat consumption, indicating that lactose-intolerant individuals did not significantly substitute animal products for dairy. It is, therefore, possible that lactose-intolerant individuals either require less dietary calcium than the non-LIIs, used more of lactose or milk derived non dairy products such as breakfast cereal [53], or that plant-based alternatives provided enough supply of calcium [47].

No significant association between calcium supplementation and BMD was found in this study, this is consistent with a previous systematic review which found that calcium intake is not significantly associated with hip fracture risk in women or men [54]. A longitudinal study of 955 post-menopausal women in London used DXA scans to measure BMD and found that calcium supplementation had no effect on bone health [55]. This may be due to the lack of vital nutrients in calcium supplements, such as thiamine, riboflavin, B-6, and B-12, that are often found in calcium-rich foods [9]. As previously mentioned, nutrients are synergistic and may absorb differently when consumed in isolation. Increasing research has also demonstrated potentially harmful side effects of calcium supplements, including gastrointestinal distress and cardiovascular disease [54,56].

It is important to note that research in favour of calcium supplementation often incorporates vitamin D supplementation into treatment [57,58,59]. Unlike calcium supplements, vitamin D has not been linked to harmful side effects (given that daily consumption does not exceed 10,000 IU). Consumption of vitamin D supplements is widely advocated especially in populations living above 37 degrees or below −37 degrees latitude [38]. A double-blind, placebo-controlled trial of postmenopausal women in Scotland found that supplements of 400 IU a day are essential to improve bone health [60]. Similarly, the current investigation found vitamin D supplements to have a positive effect on BMD of the FNH. However, vitamin D supplementation did not remain a significant predictor in the multivariate analysis.

Several potential predictors of BMD were included in a stepwise multiple linear regression analysis. However, only BMI and age were found to be significant predictors of BMD of the FNOH, FNH, and LS. In accordance with the literature [3], this study demonstrated a positive correlation between BMI and BMD of FNOH, FNH, and LS. Additionally, BMD of the FNOH, FNH, and LS was significantly lower for postmenopausal women. It is widely accepted that high BMI is protective against osteoporosis [3]. The current investigation study supports this theory.

### Strengths and Limitations

Several strengths and weaknesses result from the retrospective nature of this study. The response rate among eligible participants was 18.5% (512 out of 2770), and 496 out of 512 participants had completed survey data. Although a low response rate is common epidemiological studies, it can limit the generasibility of our findings. Recall of diet and lifestyle from the time of surgery may be inaccurate, as some surgeries occurred 14 years ago. Although the questionnaire captured intake of supplements, we did not measure the actual blood levels of calcium and vitamin D. To account for these, questionnaires were broad, capturing ranges versus precise numbers (e.g., calcium supplementation). The residential latitude at the time of the surgery was used as a proxy of sun exposure. However, individuals living in low-risk latitude can still have limited sun exposure due to cultural factors (clothing) and use of sun blocker. There are a number of factors involved in the development of osteoporosis, such as glucocorticoid therapy and hyperparathyroidism, to mention a few. However, these conditions were not considered as potential confounders in the association between lactose intolerance and BMD. Furthermore, information about all potential predictors was not available, including detailed information on ethnicity, education, and income. However, general information about the clinic profile shows that the majority of patients were white caucasians with middle and high socio-economic status. This might be due to the fact that the clinic was private. This study also relied on self-reported lactose intolerance, versus physician diagnoses or the use of a hydrogen breath test. Because symptoms of lactose intolerance were of primary interest, self-reported intolerance was likely more apt at recognizing dairy avoidance and severity of intolerance. However, without a diagnosis, it is possible that other medical conditions, such as irritable bowel syndrome, may have been mistaken for lactose intolerance.

Medical record research was also employed, which relies on the consistency and accuracy of data input [60]. However, patient medical records are still considered the gold standard in any study to identify demographic factors, clinical data variables, specific aspects related to treatment regimes, and ultimately patient mortality and morbidity [61].

Furthermore, the small sample size of lactose-intolerant individuals (*n* = 46) may lack statistical power to detect significant differences between groups. Lactose intolerant individuals may also vary greatly in their level of nutritional awareness, which may affect which food sources replace dairy items. It is expected that lactose-intolerant individuals who actively seek calcium-rich plant foods should have stronger BMDs than individuals who do not carefully plan their diet. For this reason, it is possible that the results of this study are not applicable to all dairy abstaining populations.

## 5. Conclusions

This study found that individuals with lactose intolerance were not significantly different from individuals without lactose intolerance in relation to most of the variables, although the latter group was more likely to consume vitamin D supplements and higher intake of dairy and animal protein.

In addition, there was significant variation in the mean BMD in the different BMI groups in all three indicators (FNOH, FNH, and LS) and the higher the BMI category, the higher the mean BMD. While BMI was positively associated with BMD, age was negatively associated with BMD. Furthermore, the mean BMD was not significantly different between those with or without lactose intolerance. Further research involving prospective studies and more comprehensive food frequency questionnaires are essential to confirm these findings and better understand how the consumption of dairy products is linked to low BMD.

## Figures and Tables

**Table 1:** Characteristics of patients who received hip arthroplasty between 2003 and 2017; Columbia, South Carolina, United States of America.

Categorical Variables ^a^	All Participants *n* = 496	With Lactose Intolerance *n* = 46	Without Lactose Intolerance *n* = 450	*p*-Value
**Gender**				
Male	342 (68.9%)	30 (65.2%)	312 (69.3%)	0.566 ^b^
Female	154 (21.1%)	16 (34.8%)	138 (30.7%)	
**At-risk latitude**				
Yes	145 (29.2%)	13 (28.3%)	132 (29.3%)	0.879 ^b^
No	351 (70.8%)	33 (71.7%)	318 (70.7%)	
**Smoking Status**				
Smokers	7 (1.4%)	0 (0.0%)	7 (1.6%)	0.386 ^c^
Non-smokers	489 (98.6%)	46 (100.0%)	443 (98.4%)	
**BMI categories ^d^**				0.773 ^c^
Underweight	7 (1.5%)	0 (0.0%)	7 (1.7%)	
Healthy weight	173 (37.5%)	19 (45.2%)	154 (36.7%)	
Overweight	186 (40.3%)	15 (35.7%)	171 (40.8%)	
Obese	95 (20.6%)	8 (19.1%)	87 (20.8%)	
**Menopausal status of women ^e^**				0.672 ^b^
Pre or perimenopausal	59 (38.3%)	9 (56.2%)	50 (36.2%)	
Postmenopausal	95 (61.7%)	7 (43.7%)	88 (63.8%)	
**Vitamin D Supplementation**				0.015 ^b^
Yes	117 (23.6%)	6 (13.0%)	111 (24.7%)	
No	379 (76.4%)	40 (67.0%)	339 (75.3%)	
**Calcium supplementation**				0.484 ^b^
Yes	142 (29.0%)	9 (24.3%)	133 (43.0%)	
No	354 (71.0%)	37 (75.7%)	317 (57%)	
**Consumption of food fortified with calcium** **>1 time/week**				<0.0001 ^b^
Yes	437 (88.1%)	20 (43.5%)	417 (92.7%)	
No	59 (11.9%)	26 (56.5%)	33 (7.3%)	
**Vegan or vegetarian**				0.147 ^c^
Yes	480 (97%)	3 (6.5%)	12 (2.7%)	
No	15 (3.0%)	43 (93.5%)	437 (97.3%)	
**Cardiovascular exercise at least once a week for 30 min**				0.372 ^b^
Yes	382 (77.0%)	33 (71.7%)	349 (77.7%)	
No	114 (23%)	13 (28.3%)	101 (22.4%)	
**Surgery Type**				0.724 ^b^
Hip resurfacing surgery	481 (97.0%)	45 (97.8%)	436 (96.9%)	
Total hip replacement surgery	15 (3.0%)	1 (2.2%)	14 (3.1%)	
**Osteoporosis or osteopenia**				0.123 ^b^
Yes	165 (33.3%)	20 (43.5%)	145 (32.2%)	
No	331 (66.7%)	26 (56.5%)	305 (67.8%)	
**Continuous Variables ^f^**				***p*-Value ^g^**
**Age (years)**	60.0 (0.40)	58.7 (2.27)	60.20 (0.38)	0.295
**Sum of all dairy consumption**	6.82 (0.81)	5.65 (0.28)	6.94 (0.08)	<0.0001
**Sum of all protein consumption**	12.01 (0.11)	11.04 (0.34)	12.11 (0.11)	0.004
**BMI (kg/m^2^)**	26.23 (0.21)	25.57 (0.63)	26.29 (0.23)	0.295

(**^a^**) categorical data reported as *n* (%), (**^b^**) *p*-value for Chi-Square test, (**^c^**) *p*-value for Fisher exact-test, (**^d^**) analysis was performed among 461 subjects with valid BMI measurement, (**^e^**) analysis was performed among women only (*n* = 154), (**^f^**) continuous data reported as mean and SE, (**^g^**) *p*-value for an unpaired *t*-test, BMD—bone mineral density, BMI—body mass index.

**Table 2:** Differences in bone mineral density (BMD) t-scores according to different independent variables.

Variables	BMD: Femoral Neck of the Operative Hip		BMD: Femoral Neck of the Non-Operative Hip		BMD: Lumbar Spine	
All Study Participants (*n* = 496)	Mean (SE)	*p*-Value	Mean (SE)	*p*-Value	Mean (SE)	*p*-Value
**Gender**		0.062		0.076		0.017
Males	−0.16 (0.07)		−0.10 (0.06)		1.31 (0.11)	
Females	−0.42 (0.11)		−0.30 (0.10)		0.84 (0.15)	
**Calcium supplementation**		0.202		0.707		0.325
Yes	−0.36 (1.00)		−0.13 (0.87)		1.04 (1.76)	
No	−0.18 (0.80)		−0.17 (0.64)		1.24 (1.07)	
**Vitamin D supplementation**		0.005		0.085		0.613
Yes	−0.27 (1.00)		−0.12 (1.06)		0.97 (1.91)	
No	−0.85 (1.19)		−0.55 (1.12)		0.57 (1.93)	
**Consumption of food fortified with calcium >1 time/week**		0.070		0.181		0.973
Yes	−0.19 (0.07)		−0.14 (0.05)		1.17 (0.10)	
No	−0.56 (0.18)		−0.35 (0.15)		1.16 (0.26)	
**Consumption of milk >1 time/week**		0.942		0.486		0.624
Yes	−0.23 (0.07)		−0.18 (0.05)		1.19 (0.10)	
No	−0.25 (0.14)		−0.07 (0.14)		1.06 (0.25)	
**Consumption of cheese >1 time/week**		0.434		0.700		0.274
Yes	−0.22 (0.07)		−0.16 (0.05)		1.13 (0.09)	
No	−0.38 (0.20)		−0.22 (0.15)		1.44 (0.33)	
**Consumption of yogurt >1 time/week**		0.015		0.269		0.853
Yes	−0.11 (0.08)		−0.12 (0.70)		1.16 (0.12)	
No	−0.42 (0.93)		−0.23 (0.07)		1.19 (0.14)	
**Vegan or vegetarian**		0.049		0.068		<0.001
Yes	−1.0 (0.41)		−0.69 (0.23)		−0.75 (0.52)	
No	−0.21 (0.06)		−0.15 (0.05)		1.22 (0.9)	
**At-risk Latitude**		0.103		0.652		0.643
Yes	−0.05 (0.11)		−0.13 (0.10)		1.10 (1.63)	
No	−0.29 (0.08)		−0.18 (0.61)		1.19 (1.07)	
**Menopausal status of women only ^a^**		0.0036		<0.001		0.005
Pre or perimenopausal	−0.15 (0.07)		−0.07 (0.06)		1.30 (1.65)	
Postmenopausal	−0.61 (0.13)		−0.55 (0.100)		1.540 (1.58)	
**BMI (kg/m^2^)**		<0.0001		<0.0001		<0.001
Underweight	−1.4 (0.98)		−1.30 (1.34)		−1.14 (1.35)	
Healthy	−0.54 (1.16)		−0.46 (1.07)		0.85 (1.75)	
Overweight	−0.16 (1.16)		−0.11 (1.11)		1.23 (1.79	
Obese	0.28 (1.08)		0.29 (1.09)		1.624 (1.56)	
**Cardiovascular exercise at least once a week for 30 min**		0.546		0.133		0.753
Yes	−0.21 (0.07)		−0.12 (0.06)		1.15 (0.10)	
No	−0.30 (0.14)		−0.31 (0.11)		1.22 (0.17)	

(**^a^**) analysis was performed among women only (*n* = 154); BMD—bone mineral density, BMI—body mass index.

**Table 3:** Differences in bone mineral density (BMD) t-scores according to lactose intolerance status.

BMD (T-Scores)	All Participants *n* = 496 Mean (SE)	With Lactose Intolerance *n* = 46 Mean (SE)	Without Lactose Intolerance *n* = 450 Mean (SE)	*p*-Value
**BMD: Femoral neck of the operative hip**	−0.23 (0.63)	−0.30 (0.22)	−0.23 (0.65)	0.766
**BMD: Femoral neck of the non-operative hip**	−0.16 (0.05)	−0.23 (0.17)	−0.16 (0.05)	0.698
**BMD: Lumbar spine**	1.17 (0.09)	1.19 (0.33)	1.17 (0.09)	0.954

BMD—bone mineral density.

**Table 4:** Predictors of Bone Mineral Density according to a stepwise linear regression model.

Variables	BMD: Femoral Neck of the Operative Hip		BMD: Femoral Neck of the Non-Operative Hip		BMD: Lumbar Spine	
	**β (SE)**	***p*-Value**	**β (SE)**	***p*-Value**	**β (SE)**	***p*-Value**
BMI (kg/m^2^)	0.06 (0.01)	<0.0001	0.07 (0.11)	<0.0001	0.06 (0.02)	0.008
Age (years)	−0.16 (.01)	0.047	−0.02 (.01)	0.001	−0.03 (0.01)	0.004
Yogurt >1 time/week	0.33 (0.13)	0.014				
Cardiovascular exercise >1 time/week			0.35 (0.12)	0.005		
Gender (males)					0.43 (0.21)	0.04
Vegan or Vegetarian					−1.26 (0.59)	0.33
Adjusted R^2^	7.4%		10.3%		7.3%	

BMD—bone mass density.

## Abbreviations

DALY	disability-adjusted life years
DXA	dual-energy X-ray absorptiometry
BMD	bone mineral density
SD	standard deviations
WHO	World Health Organization
LIIs	lactose intolerant individuals
FNOH	femoral neck of the operative hip
FNH	femoral neck of the non-operative hip
LS	lumbar spine
BMI	body mass index
ARL	at risk latitude
NRL	no risk latitude
PBH	poor bone health
ANOVA	one-way analysis of variance
